# In vitro approaches to investigate the effect of chemicals on antibody production: the case study of PFASs

**DOI:** 10.1007/s00204-025-03993-6

**Published:** 2025-03-06

**Authors:** Martina Iulini, Valeria Bettinsoli, Ambra Maddalon, Valentina Galbiati, Aafke W. F. Janssen, Karsten Beekmann, Giulia Russo, Francesco Pappalardo, Styliani Fragki, Alicia Paini, Emanuela Corsini

**Affiliations:** 1 Laboratory of Toxicology, Department of Pharmacological and Biomolecular Sciences “Rodolfo Paoletti”, Università degli Studi di Milano, Via Balzaretti 9, 20133 Milan, Italy; 2 Department of Pharmacy, Università degli Studi di Napoli Federico II, Napoli, Italy; 3 Wageningen Food Safety Research (WFSR), Wageningen, The Netherlands; 4 Department of Health and Drug Sciences, Università degli Studi di Catania, Catania, Italy; 5 esqLABS GmbH, 26683 Saterland, Germany

**Keywords:** Antibody production, Immunosuppression, NAM, PFASs

## Abstract

The increasing variety and quantity of new chemical substances have raised concerns about their potential immunotoxic effects, making it essential to assess their impact on human health. One key concern is the reduction of antibody production, as seen with per- and poly-fluoroalkyl substances (PFASs), commonly known as “forever chemicals.” Both in vivo and epidemiological data show that PFASs have immunosuppressive effects, leading to reduced antibody responses, particularly following vaccination. In animal studies, the T cell-dependent (TD) antibody response is the gold standard for assessing chemical effects on immune function. This study utilized two in vitro approaches to investigate the effects of chemicals on antibody production using human peripheral blood mononuclear cells. Initial tests used unstimulated, negative (vehicle), and positive (rapamycin) controls to confirm the robustness of the models. Subsequently, four long-chain PFASs (PFOA, PFOS, PFNA, and PFHxS) were tested. Keyhole limpet hemocyanin (KLH) was used to mimic the TD response, while a TLR9 agonist and IL-2 activated B cells for T cell-independent (TI) immunoglobulin production. The results demonstrated the ability to reproduce TD and TI responses in vitro with robust, reproducible outcomes across a cohort of 20 human donors. The data, consistent with existing literature, showed a significant reduction in anti-KLH IgM production, especially for PFOA in male donors. Similar trends were observed for all PFASs in suppressing total TI IgG and IgM production. These methods closely replicated in vivo conditions, offering a potential alternative to animal models in immunotoxicity assessments.

**Supplementary Information:**

The online version contains supplementary material available at 10.1007/s00204-025-03993-6.

## Introduction

A well-functioning immune system is essential for maintaining the integrity of the organism. Immune cells are seamlessly integrated into various systems and consequently, exposure to immunotoxic substances can impair the body’s ability to mount an effective response. Immunotoxic agents can lead to immune system suppression, reducing the ability of the body to defend against bacterial, viral, and fungal infections effectively. Additionally, certain substances may trigger abnormal immune responses, increasing the risk of autoimmune diseases and allergic reactions, especially in sensitive individuals. The severity of these effects depends on the specific chemicals, as well as the duration and intensity of exposure. Understanding the immunotoxic potential of chemicals is essential to assess associated health risks. Functional immunotoxicity testing, except for skin sensitization, is rarely conducted on new chemicals (Ehrlich et al. 2023). Given the health risks associated with exposure to immunotoxic compounds and the increasing variety and quantity of new chemicals thorough immunotoxicity investigations are warranted. Currently, immunotoxicity assessment primarily relies on animal tests. In this context, given the ongoing shift in the approach to toxicity assessment, increasing endeavors are being directed towards replacing these conventional techniques with new approach methodologies (NAMs). In the vision of toxicology in the twenty-first century, animal-free testing techniques promote the development and validation of strategies that ensure chemical safety without relying on animal experiments (Westmoreland et al. 2022).

Diverging from the conventional in vivo tests employed to explore systemic chemical toxicity, in vitro tests are applied to get insight into the underlying mode of action. It is believed that in vitro assays for screening purposes typically should involve multiple assessments to detect immunotoxic substances due to the distinct components of the immune system and their interactions with other organs. Among chemical-induced immunotoxicity, one of the outcomes is immunosuppression. It can manifest with alteration of cellularity, cytokines, chemokine, antibody, and growth factor production, as well as alteration in specific lymphocyte subpopulations and their function (OECD 2022). In this paper, we describe a strategy to assess in vitro antibody production, as T cell-dependent antibody production (TDAR) is recognized in vivo as the most sensitive endpoint to identify immunotoxic compounds (Luster et al. 1992).

The immune system can trigger the release of antibodies from B cells in two ways: through T cell-dependent (TD) or T cell-independent (TI) mechanisms. In TD B cell activation, three types of cells are involved. Antigens are recognized and presented by antigen-presenting cells (APCs) to naïve T cells via an MHC class II-mediated process. After antigen uptake, APCs increase the expression of class II molecules and co-stimulatory factors like CD80 and CD86. T cells activated by APCs then signal B cells through the interaction between the T cell CD40 ligand (CD40L) and the CD40 receptor on B cells, along with the release of associated cytokines. This CD40L-CD40 interaction leads to the production of antibodies, specifically immunoglobulin (Ig) G, IgA, IgE, and IgM. Additionally, CD40 signaling is essential for isotype switching, which is crucial for developing B cell memory—a key factor in the success of vaccinations (Chaplin 2010). Alternatively, antibodies can be produced through a TI B cell response. In this case, B cells directly recognize antigens via binding to the B cell receptor and engagement with specific toll-like receptors (TLRs), such as TLR-4 and TLR-9. Following this interaction, B cells differentiate into plasma cells capable of secreting antibodies (Pone et al. 2015). While the TI B cell response is shorter in time than the TD response and does not result in affinity-matured antibodies, it can, in certain cases, lead to long-term antibody production (Bortnick et al. 2012).

In line with the global vision of toxicology in the twenty-first century, our objective was to develop in vitro approach for enhancing the comprehensive assessment of chemical risks assessment. We used per- and polyfluoroalkyl substances (PFASs) as a case study. PFASs are a broad class of man-made organofluorine chemicals composed of an aliphatic carbon chain in which the hydrogen atoms are replaced by fluorine atoms. For our research, we used four of the most common PFASs found in human plasma, namely perfluorooctane sulfonate (PFOS), perfluorooctanoic acid (PFOA), perfluorononanoic acid (PFNA) and perfluorohexane sulfonic acid (PFHxS) (EFSA CONTAM PANEL et al. 2020; Sun et al. 2018). Human epidemiological studies revealed that a doubling of PFOS in maternal serum was associated with a reduction of 39% (*p* < 0.001) in diphtheria antibody concentration in children of 5 years and that reduction increased the chances of falling in protective values against diphtheria and tetanus at age 7 years (Grandjean et al. 2012). The same paper also reported that an increased concentration (twofold change) of the major PFASs in the children’s serum was associated also with a reduction in the overall antibody concentration (Grandjean et al. 2012). The same cohort was examined in the adolescence period and a decrease in immunological response was found also at the age of 13 years (Grandjean et al. 2017). Reduction in the antibody response was also noted for the vaccination against rubella, mumps, and Hemophilus influenza in children and adult (Abraham et al. 2020; Stein et al. 2016; Looker et al. 2014; Granum et al. 2013). The correlations between levels of PFASs in blood/serum and the reduced effectiveness of vaccinations observed in human studies suggest that PFASs may affect human immune responses. Different animal studies reported the correlation between immunomodulation and higher levels of PFASs in the blood (Peden-Adams et al. 2008; Qazi et al. 2010; Zheng et al. 2009). Due to the different PFASs half-lives and different immune systems between humans and animals, animal studies do not represent completely the point of departure to study human risk characterization (Antoniou et al. 2022). This highlights the need to develop a robust human-based in vitro model that accurately reflects human biology, enabling a deeper understanding of PFASs immunotoxicity, particularly its impact on the human immune system in vitro. Such a model would not only enhance our knowledge of the mechanisms driving PFAS-related immunotoxicity but also contribute to more accurate risk assessments for human health.

## Materials and methods

**Chemicals**: PFOA (CAS #335-67-1, purity 95%), PFOS (CAS #1763-23-1, acid solution ~ 40% in H_2_O), PFNA (CAS #375-95-1), PFHxS (CAS #381-99-6) and rapamycin (CAS # 53123-88-9) were purchased from Sigma-Aldrich (St Louis, MO, United States). All the chemicals were diluted in dimethyl sulfoxide (DMSO, CAS #67–68-5). The final concentration of DMSO in cell culture was 0.1%. KLH (Keyhole limpet hemocyanin; CAS# 9013-72-3) and SAC (*Staphylococcus aureus* Cowan I; PANSORBIN® Cells) were purchased from Sigma-Aldrich, and rhIL-2 was purchased from Miltenyi Biotec (Bergisch Gladbach, Germany), while ODN2006 (ODN 7909) was purchased from InvivoGen (San Diego, CA, United States). KLH, SAC, rhIL-2, and ODN2006 were diluted in Dulbecco’s phosphate-buffered saline (PBS).

### Cells

PBMCs were isolated from anonymous buffy coats obtained from male and female healthy donors purchased from the Niguarda Hospital (Milan, Italy). PBMCs were isolated by Ficoll gradient centrifugation and subsequently washed 5 times with PBS. For the experiments, PBMCs were suspended in RPMI 1640 without phenol red containing 2 mM L-glutamine, 100 IU/mL penicillin, 0.1 mg/mL streptomycin, 10 µg/mL gentamycin, 50 µM 2-mercaptoethanol, supplemented with 5% of heat-inactivated human serum (complete medium). Cell culture medium, serum, and all supplements were purchased from Sigma-Aldrich. Cells were treated with increasing concentrations of PFOA, PFOS, PFNA and PFHxS, rapamycin, and DMSO as vehicle control. Before proceeding with the treatments, an initial experiment was performed to identify the non-cytotoxic concentrations of PFASs. For the determination of the leukotoxicity, the CyQUANT™ LDH Cytotoxicity Assay Kit was used (InvitrogenTM Corporation, Massachusetts, US) and the manufacturer’s procedures were followed. The results are reported in the Supplementary materials Fig. 1. The selected concentration range, which includes values from ng/mL to µg/mL, reflects the evidence that median serum levels of PFOA and PFOS in the general population typically fall between 2–10 ng/mL but can reach significantly higher levels, up to the µg/mL range, in individuals residing near contaminated areas or working in occupational settings (Borghese et al. 2024; Gebbink and van Leeuwen 2020; Sun et al. 2018) Moreover, these concentrations are associated with exposure to contaminated water, food sources, and consumer products (Borghese et al. 2024).

**Fig. 1:**
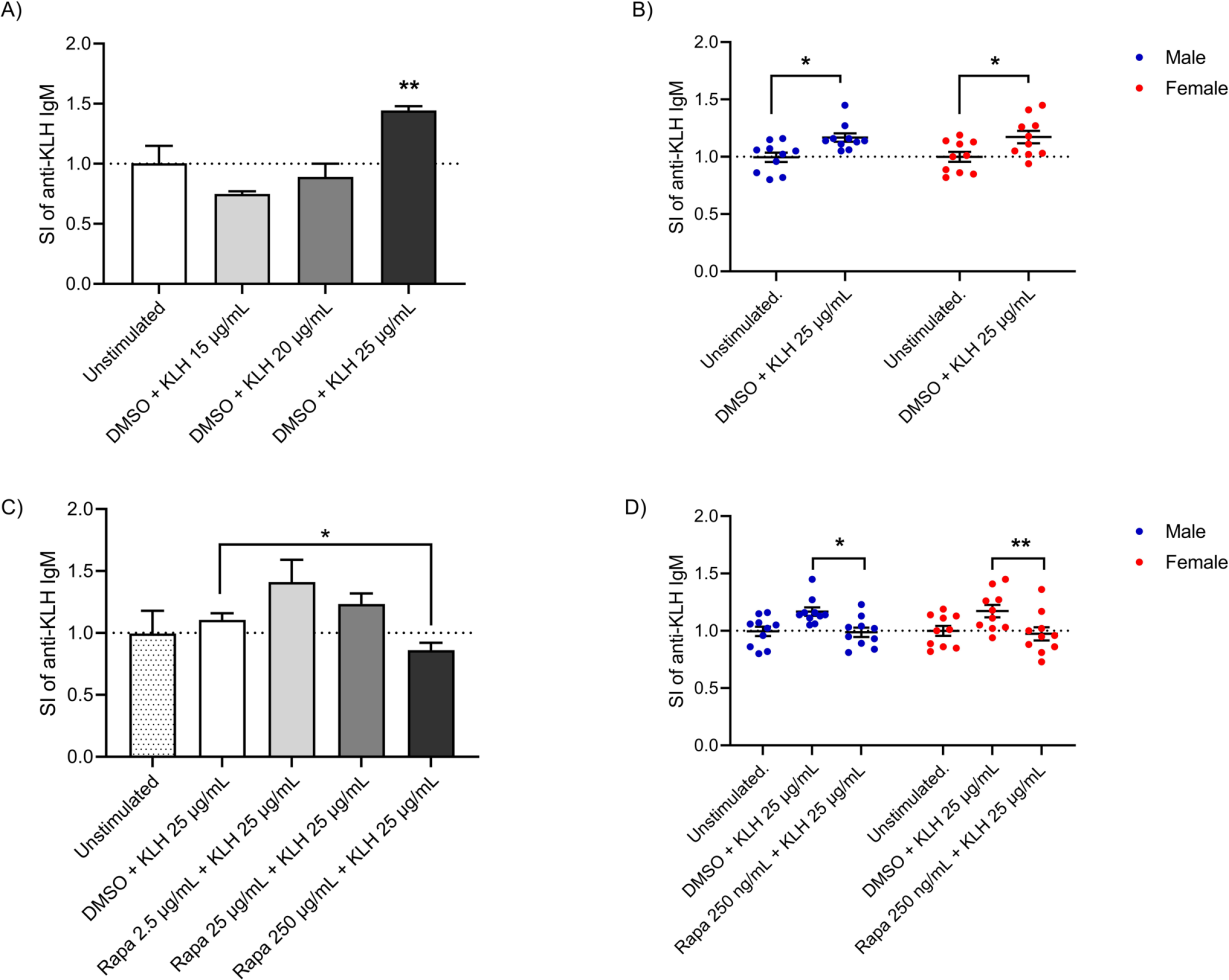
Establishment and performance of the TD antibody production. **A** identification of the optimal KLH concentration. **B** anti-KLH IgM production in male and female donors in response to KLH (25 µg/mL). **C** identification of the optimal rapamycin concentration. **D** assessment of the protocol using unstimulated and positive controls (rapamycin 250 ng/mL). Results are expressed as SI of IgM anti-KLH compared to unstimulated control. Each value represents the mean ± SEM, with *n* = 2 males and *n* = 2 females (**A**, **C**), and *n* = 10 males and *n* = 10 females (**B**, **D**). Experiments shown in Figure (**B**, **D**) were conducted over two years. Statistical analysis was carried out using one-way ANOVA, followed by Dunnett’s test (**A**, **B**), two-way ANOVA multiple comparisons (**B**) and unpaired t test (**D**). Results were considered significant if *p* ≤ 0.05, with * *p* ≤ 0.05 and ** *p* ≤ 0.01

### TD antibody production

#### Cell treatment

PBMCs (2.5 × 10^6^ cells/mL) were seeded in 24-well plates in a complete medium. Cells were exposed to increasing concentrations of the four selected PFASs (0.0025, 0.025, 0.25, 2.5 and 25 µg/mL), rapamycin (250 ng/mL) or DMSO as vehicle control and incubated at 37 °C in 5% CO_2_ for 24 h and then stimulated or not with 25 µg/mL of KLH and 30 µg/mL of SAC for 5 days. After a total of 6 days, the medium was changed, cells were washed twice with PBS and then resuspended in fresh complete medium. Cells were treated again with PFOA, PFOS, PFNA, PFHxS, rapamycin or DMSO in the presence of 60 IU/mL of rhIL-2. The cells were incubated for an additional 5 days at 37 °C in 5% CO_2_.

##### Anti KLH-IgM measurement

To evaluate the release of anti-KLH antibodies, PBMCs were harvested and centrifuged for 5 min at 25 °C at 2000 rpm. The resulting supernatants were collected and stored at −20 °C until measurement. The release of human IgM anti-KLH was assessed using a commercially available ELISA kit (Cusabio, Houston, TX, United States; CSB E16534h). Assays were performed following the manufacturer’s instructions. To determine ODs, the Molecular Devices SpectraMax ABS spectrophotometer was used. Data were collected and analyzed by integrated software SoftMax Pro 7.1.2. Results are expressed as fold-change of chemicals-treated cells *versus* vehicle-treated cells.

A more accurate and detailed explanation of the entire protocol can be found in the standard operation procedures section of Corsini et al. (2024).

### TI antibody production

#### Cell treatment

PBMCs (1.26 × 10^6^ cells/mL) were seeded in 48-well plates in a complete medium. Cells were treated with increasing concentrations of PFOA, PFNA, PFHxS, and PFOS (0.00126, 0.126, and 12.6 µg/mL), rapamycin (2.52 ng/mL) or DMSO as vehicle control and incubated at 37 °C in 5% CO_2_ for 24 h. PBMCs were then stimulated or not with 1 µg/mL of ODN2006 and 100 IU/mL of rhIL-2 for 5 days.

#### IgG and IgM release

To determine the release of total IgG and IgM, after a total of 6 days, PBMCs were harvested and centrifuged for 5 min at 25 °C at 2000 rpm. Supernatants were collected and stored at −20 °C until measurement. IgM and IgG or total Ig (IgG + IgM) release was evaluated using a custom ELISA that was assembled in-house from individual reagents all purchased from Sigma-Aldrich. Briefly, 100 µL of anti-human IgG (Cat. No. I1886) and/or anti-human IgM (Cat. No. I0104) solution at 1 µg/mL in PBS were plated in a 96-well plate and incubated overnight at 4 °C. After that, 100 µL of standard (0–1000 ng/ml) or samples diluted in reagent buffer (PBS + 0.5% of bovine serum albumin + 0.05% of Tween 20) were plated and incubated for 2 h at room temperature. Then, 100 µL of anti-human polyvalent Igs (Cat. No. A3313) diluted 1:2000 in reagent buffer were added and incubated for 1 h at room temperature. At the end, 100 µL of Sigma 104 substrate diluted in AP buffer (Cat. No. A4955) was added to the plates. Absorbance was read at 415 nm. Data were analyzed by integrated software SoftMax Pro 7.1.2. Results are expressed as fold-change of chemicals-treated cells *versus* vehicle-treated cells.

A more accurate and detailed explanation of the entire protocol can be found in the standard operation procedures section of Corsini et al. (2024).

### Statistical analysis

Results are expressed as mean ± standard error of the mean (SEM). All the results are expressed as a stimulation index (SI) obtained by the ratio between the treated condition and its respective internal control. For Fig. 1, the SI was calculated using the unstimulated control to highlight the stimulation effect. For the remaining figures, the SI was normalized to the vehicle control (concentration 0 µg/mL) to account for baseline effects. Statistical analysis was performed using GraphPad Prism version 10.2.3 (GraphPad Software, La Jolla, CA, United States). Significant differences were determined using paired or unpaired T-test, or analysis of variance (ANOVA), followed, when significant, by an appropriate post hoc test, as indicated in the Figure legends. Effects were designated as significant if the *p* value was ≤ 0.05.

## Results

### Establishment and assessment of the TD antibody production protocol

It is notoriously difficult to obtain a primary response to an exogenous antigen in a simple culture of PBMCs, due to the low frequency of naïve T and B cells. To reproduce the primary response, we modified the method developed by Komatsu et al. (1986) with the addition of IL-2 to boost the activation of cells (Kennell et al. 2014). To begin, we conducted preliminary tests to assess the feasibility of the protocol, optimizing the concentration of KLH for our specific conditions and confirming the suppression efficacy of the positive control rapamycin. In brief, to select the best concentration for KLH, PBMCs were exposed to different concentrations of KLH (15, 20 or 25 µg/mL) in the presence of SAC at a concentration of 30 mg/mL for 5 days. Thereafter, cells were washed and further cultured in the presence of rhIL-2 (60 IU/mL) for an additional 5 days before to assess the release of anti-KLH IgM. Starting from the results reported in Fig. 1A, B, the selected concentration was 25 µg/mL for KLH. After the selection of the best induction, we performed experiments to choose the proper concentration for the positive control rapamycin. PBMC were treated for 24 h with 2.5, 25 or 250 ng/mL of rapamycin, a mTORC1 inhibitor. Cells were then exposed to 25 µg/mL of KLH in the presence of SAC at a concentration of 30 mg/mL for 5 days. Thereafter, cells were washed and further cultured in the presence of rhIL-2 (60 IU/mL) and rapamycin for an additional 5 days. The results are reported in Fig. 1C, D.

In Fig. 1 the establishment and performance of the TD antibody response method is reported. In Fig. 1A, preliminary experiments were conducted to establish optimal KLH concentrations. Three increasing KLH concentrations (15, 20, 25 µg/mL) were tested. Under our experimental conditions, only the concentration of 25 µg/mL was able to induce a statistically significant increase in anti-KLH IgM release compared to the unstimulated control. In Fig. 1B, the antibody response obtained over 2 years in males and females is reported. The antibody response to KLH was evaluated using a total of 10 male and 10 female donors to assess the consistency of the response, donor variability, and potential sex differences. While all donors tested responded to KLH with the production of specific antibodies, the induction of IgM anti-KLH release upon stimulation with SAC and IL-2 appeared to be highly variable among donors. Although this, a comparable response was obtained in males and females. In Fig. 1C, the selection of the best concentration for rapamycin is reported. In Fig. 1D, the response to the chosen positive control rapamycin (250 ng/mL) in both male and female donors is reported. A consistent statistically significant reduction was observed in both male and female donors, with no sex differences. Once the experimental conditions were established, as proof of principle well-known immunosuppressive environmental contaminants (PFOA, PFOS, PFNA, and PFHxS) were tested. It is important to mention that a limitation of this study is the lack of information on the PFAS levels in the serum of the donors, which may contribute to the observed variability in immune responses, especially in female donors, where 4 of 10 showed low response.

### Effect of PFOA, PFNA, PFHxS and PFOS on TD antibody production

PBMCs were treated for 24 h with the four selected PFAS at increasing concentrations (0.0025, 0.025, 0.25, 2.5 and 25 µg/mL). The selection of a wide range of concentrations was made to include both low levels (ng/mL), relevant to the general population, and higher levels (µg/mL), pertinent to highly exposed workers. This allowed us to identify effects, and their relevance to humans. The cells were then exposed to 25 µg/mL of KLH in the presence of SAC (30 mg/mL) for 5 days. Thereafter, cells were washed and further cultured in the presence of rhIL-2 (60 IU/mL), PFASs for an additional 5 days. In the end, the specific anti-KLH IgM antibody ELISA kit was used to assess the effect of the four PFAS on the primary antibody response.

**Fig. 2:**
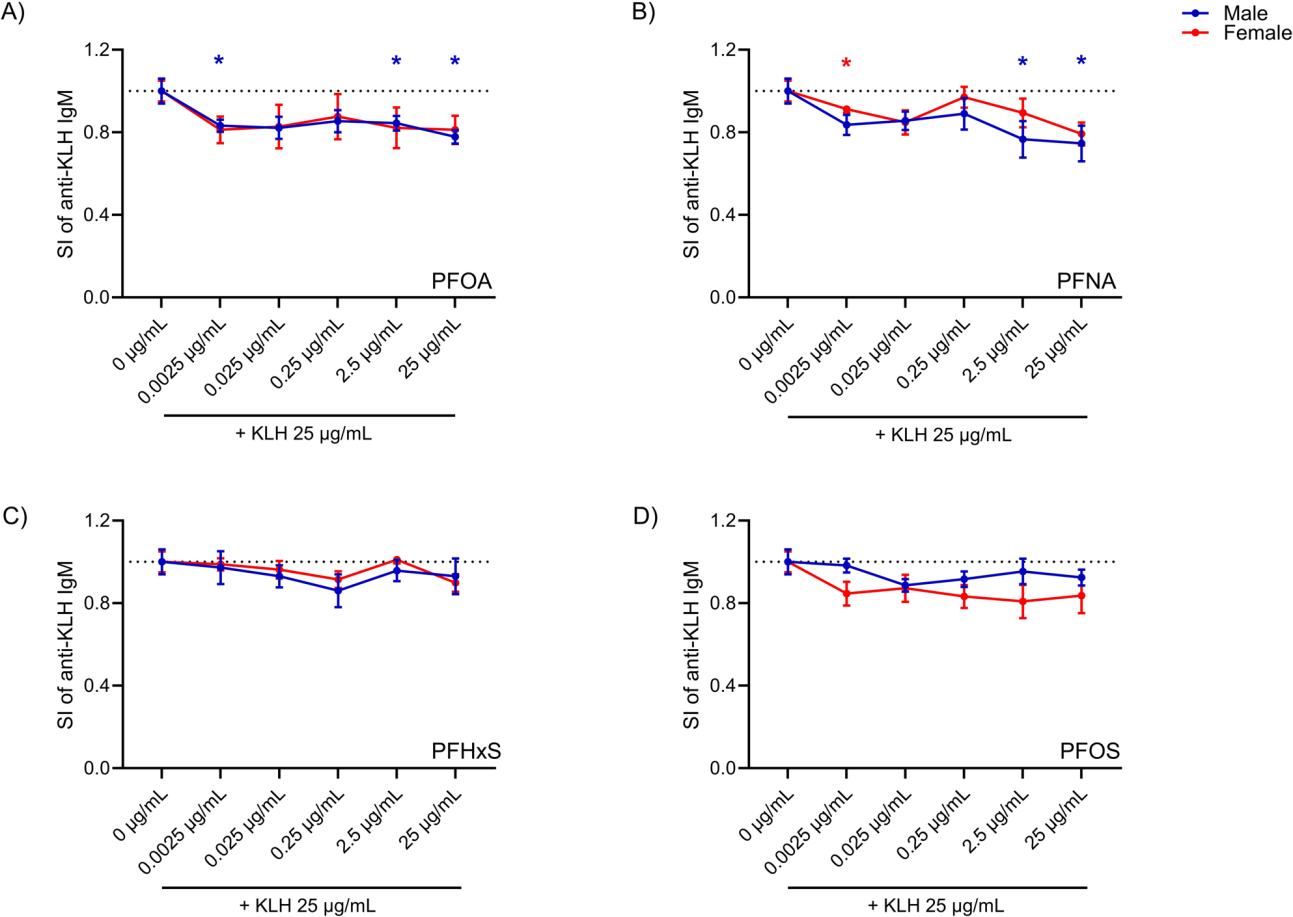
Effect of PFOA, PFNA, PFHxS, and PFOS on the production of anti-KLH IgM. The release of specific anti-KLH IgM after PFOA (**A**), PFNA (**B**), PFHxS (**C**) and PFOS (**D**) treatment in male (represented by the color blue) and female (represented by the color red) donors are shown. PBMCs (2.5 × 10^6^ cells/mL) were treated for 24 h with increasing concentrations of the selected PFAS (0.0025, 0.025, 0.25, 2.5 and 25 µg/mL). Cells were then stimulated with KLH and SAC. After 5 days the medium was changed and cells were incubated in the presence of PFAS and a booster of rhIL-2 for another 5 days. In the end, the release of specific anti-KLH IgM was analyzed by ELISA. The results are expressed as SI of IgM anti-KLH. Each value represents the mean ± SEM, with *n* = 5 males and *n* = 5 females. Statistical analysis was carried out using one-way ANOVA, followed by Dunnett’s test for PFAS vs Ctrl. Results were considered significant if *p* ≤ 0.05, with * *p* ≤ 0.05 vs control negative (0 µg/mL). The color of the asterisks matches the color used to indicate different sexes (color figure online)

In Fig. 2 the effects of PFOA (2A), PFNA (2B), PFHxS (2C), and PFOS (2D) on the primary antibody response to KLH for male and female donors are reported. Due to the variability in responses among donors and the limited number of donors tested (*n* = 5), a trend toward a decrease in the response to all PFAS was observed in both males and females. A statistically significant inhibition of anti-KLH IgM production was observed in males following exposure to PFOA at the concentrations of 0.0025, 2.5, and 25 µg/mL and PFNA at 2.5 and 25 µg/mL, and in females treated with 0.0025 µg/mL of PFNA. For this parameter, among the four PFAS tested, PFOA appeared to be the most potent, while PFHxS was the least active in both sexes.

Overall, the method was suitable to identify immunotoxic compounds. The observed reduction in antibody response with all PFAS tested is in agreement with the in vivo findings (Crawford et al. 2023; De Guise and Levin 2021).

### Establishment and assessment of the TI immunoglobulin production protocol

To mimic TI antibody production, the protocol described by Tuijnenburg et al. (2020) was used. This assay was developed to screen new drugs targeting B cells that may be useful in autoimmune diseases (Tuijnenburg et al. 2020). Initial experiments were conducted to establish and validate the method by assessing consistency in the response and adequate suppression by the positive control rapamycin (2.52 ng/mL). In brief, PBMCs were cultured for 24 h with rapamycin and then stimulated with 1 µg/mL ODN2006 and with 100 U/mL rhIL-2 for 6 days to induce Ig production. ODN2006, a TLR9 agonist, was used as an efficient inducer of B cell proliferation and survival. As a readout, IgM and IgG were quantified using ELISA.

**Fig. 3:**
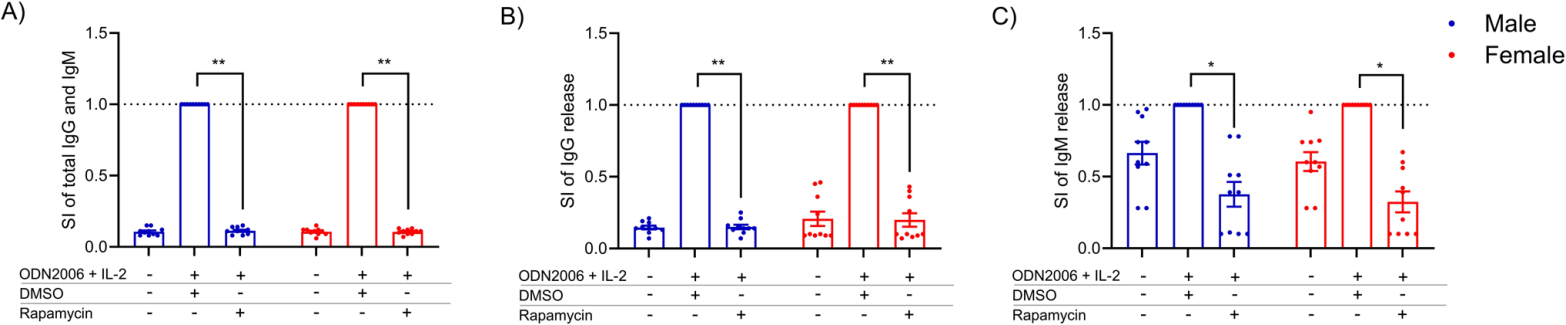
Assessment of the TI immunoglobulin production. Analysis of the release of total IgG and IgM (**A**), IgG release (**B**) and IgM release (**C**) after the treatment with rapamycin in male and female human donors. Human PBMCs were treated for 24 h by vehicle (DMSO) or rapamycin (2.52µg/mL). Then, cells were or were not stimulated with 1 µg/mL of ODN2006 and 100 U/mL of IL-2 to induce the B cell differentiation. After 6 days, the release of IgG and IgM were analyzed. The results are expressed as SI of IgG and IgM respect to vehicle-treated cells. Each value represents the mean ± SEM, with *n* = 10 female and 10 male donors. Statistical analysis was carried out using an unpaired t test for rapamycin vs DMSO. Results were considered significant if *p* ≤ 0.05, with *p < 0.05, **p < 0.01 vs Ctrl

In Fig. 3, the stimulation index (SI) of antibody release in response to ODN2006 + IL-2, with or without rapamycin and DMSO (used as solvent control) are reported. The results are stratified by sex, with blue dots representing male donors and red dots representing female donors. In Fig. 3A total Igs (IgG + IgM) are shown, while IgG and IgM are shown in Fig. 3B, C, respectively. Rapamycin significantly suppresses ODN2006 + IL-2- induced immunoglobulins release to a similar extent in both male and female donors (with an average reduction comparable between males and females, at 89% and 90%, respectively.), demonstrating its potent immunosuppressive effects. A statistically significant reduction in IgG and IgM release was consistently observed with the positive control rapamycin, with an average reduction of 90% for total IgG and IgM, an 85% decrease for IgG and 70% for IgM, in both sexes.

Once the experimental conditions were established, as proof of principle well-known immunosuppressive environmental contaminants (PFOA, PFOS, PFNA, PFHxS) were tested.

### Effect of PFOA, PFNA, PFHxS and PFOS on TI Ig release

The effects of the four selected PFAS were then evaluated according to the method described above.

**Fig. 4:**
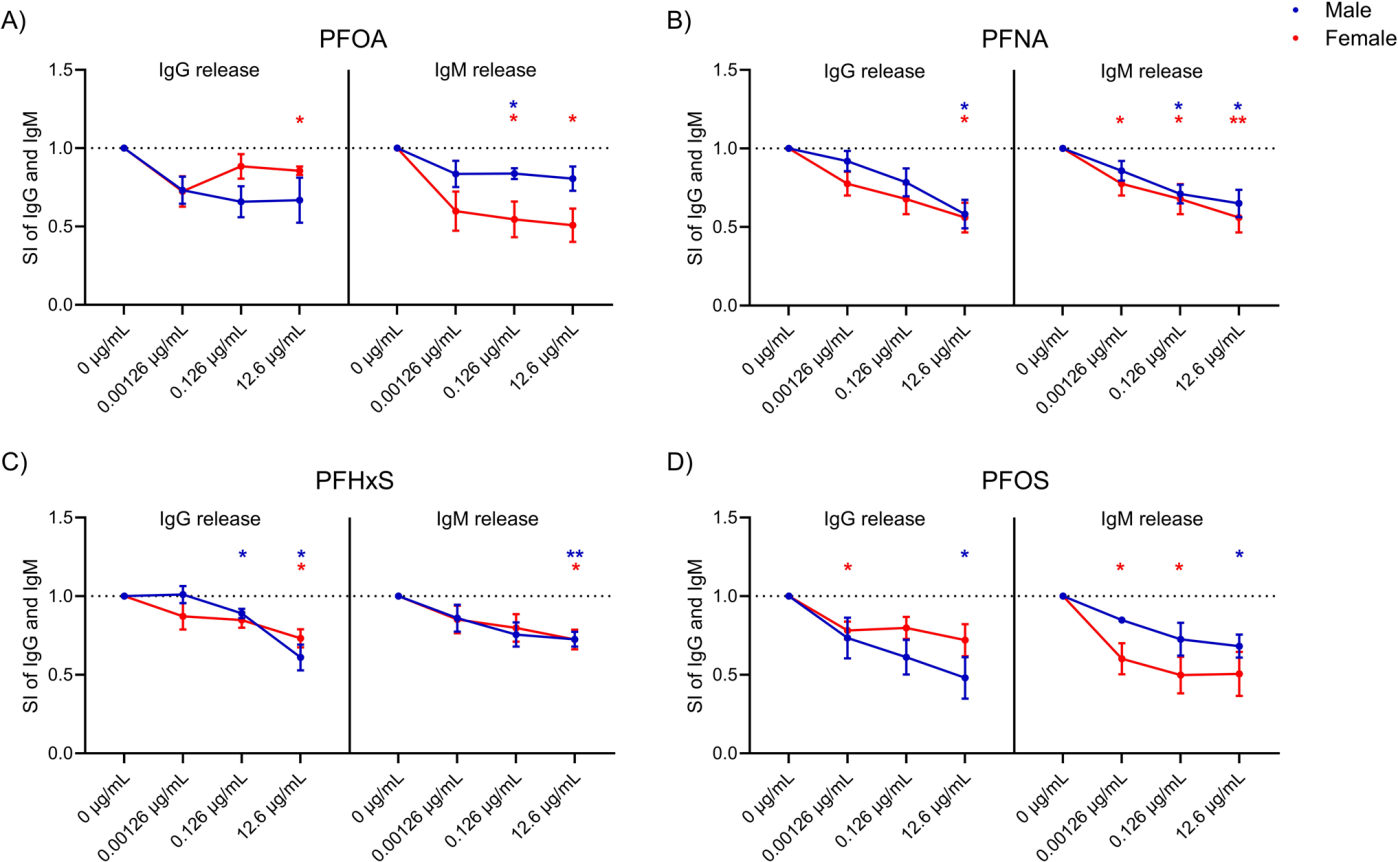
Effect of the PFOA, PFNA, PFHxS, and PFOS on IgG and IgM release. Analysis of IgG and IgM release after the treatment with PFOA (**A**), PFNA (**B**), PFHXS (**C**), and PFOS (**D**) in male (represented by the color blue) and female (represented by the color red) human donors. Human PBMCs were exposed to increasing concentrations of PFAS (0.00126, 0.126, and 12.6 µg/mL) for 24 h. Then, cells were stimulated with 1 µg/mL g/mL of ODN2006 and 100 U/mL of IL-2 to induce the B cell activation. After 6 days, the release of IgG and IgM were analyzed. The results are expressed as SI of IgG or IgM with respect to vehicle-treated cells. Each value represents the mean ± SEM, with *n* = 5 female and 5 male donors. Statistical analysis was carried out using one-way ANOVA, followed by Dunnett’s test for PFAS vs Ctrl. Results were considered significant if *p* ≤ 0.05, with *p < 0.05, **p < 0.01 vs Ctrl. The color of the asterisks matches the color used to indicate different sexes (color figure online)

In Fig. 4, the effects of PFASs on the release of IgG and IgM in male and female donors are reported. Overall, all four PFASs were able to reduce the release of both IgG and IgM in a concentration-related manner, with the exception of IgG release in female donors. The highest concentration (12.6 µg/mL) of PFOA (Fig. 4A), PFNA (Fig. 4B) and PFHxS (Fig. 4C) were able to statistically significantly suppress the release of both IgG and IgM in female donors, while the same concentration was able to suppress the release of both IgG and IgM in male donors after PFNA (Fig. 4B), PFHxS (Fig. 4C) and PFOS (Fig. 4D) exposure. Although not statistically significant, a sex difference in the release of individual immunoglobulins can be observed, with IgM release being more suppressed in female donors compared to males, particularly for PFOA (Fig. 4A) and PFOS (Fig. 4D). Additionally, PFNA (Fig. 4B) and PFOS (Fig. 4D) significantly decreased IgM release at the lowest concentration tested (0.00126 µg/mL) in female donors, indicating that even lower concentrations were able to impact antibody production.

To assess potential differences in potency, the concentration required to inhibit 20% of the response compared to the control (IC_20_) was calculated based on the total release of IgG and IgM combined, as shown in Table 1. It is possible to appreciate statistically significant differences in the IC_20_ values between male and female donors, with females being more susceptible. Considering both sexes together, the ranking of potency of reducing IgM/IgG secretion calculated in the table follows the order of PFOA > PFOS > PFNA > PFHxS.

**Table 1:** IC_20_ values of PFAS-inhibition of IgM + IgG release

	Males and females IC_20_ (µg/mL)	Males IC_20_ (µg/mL)	Females IC_20_ (µg/mL)
PFOA	2.432 ± 1.616	4.851 ± 2.971	0.012 ± 0.007 **
PFNA	4.679 ± 1.927	6.826 ± 2.840	2.531 ± 2.517*
PFHxS	6.812 ± 1.989	12.6 ± 0.001	1.024 ± 1.023**
PFOS	2.543 ± 1.678	2.526 ± 2.519	2.543 ± 2.514

Each value represents the mean ± SEM, with *n* = 5 female and 5 male donors. Statistical analysis was carried out using unpaired *t* test for male vs female. Results were considered significant if *p* ≤ 0.05, with *p < 0.05 **p < 0.01

## Discussion

The “gold standard” for identifying immunosuppressive chemicals in immunotoxicological studies is the TDAR (Luster et al. 1992; Lebrec et al. 2014). With this study, we aimed to develop simple and reproducible in vitro methods to assess the effect of chemicals on TD and TI antibody responses. To maximize relevance to humans and avoid inter-species extrapolation, human PBMCs from both male and female healthy donors were used. This approach allows for the examination of potential sex differences and reflects the variability in individual immune responses. To verify the methods, PFASs were selected as reference compounds due to their ability to suppress the antibody production in both animals and humans (Peden-Adams et al. 2008; Crawford et al. 2023).

In the standard TDAR experiment, rodents are exposed to tested chemicals and then immunized with sheep red blood cells (SRBC) or KLH to assess the antibody response (Vandebriel et al. 2024). The possibility to obtain in vitro a primary antibody response has been challenging due to low frequency in naïve B cells. Many years ago, an in vitro antibody response, also known as Mishell-Dutton culture, was developed (Mishell and Dutton, 1967). The method foresees the use of mouse spleen cells and 5 days of treatment in the presence of chemicals and SRBC, then complement is added to lyse SRBC in the antibody-secreting regions, resulting in hemolysis plaques, which represent antibody-forming cells (Mishell and Dutton, 1967; Fischer et al. 2011; Koeper and Vohr 2009). To obtain a primary response to an exogenous antigen in a simple culture of PBMCs, we adapted the protocol developed by Komatsu et al. (1986). The authors used KLH as an antigen to study the antigen-specific antibody release in nonadherent cells derived from human PBMCs. We added IL-2 as a booster for immune cell activation, as suggested by Kennell et al. (2014). Following the same process and using the same cohort of donors (10 male and 10 female donors), we also successfully confirmed the reproducibility and robustness of the TD protocol. Results obtained demonstrate the feasibility of measuring primary antibody responses in vitro using human PBMCs. The observed reduction in antibody production following PFASs exposure, particularly PFOA and PFOS, aligns with findings from epidemiological human studies and in vivo animal research (Grandjean et al. 2012; Kielsen et al. 2016; De Guise and Levin 2021; Taylor et al. 2023), confirming the effectiveness of our in vitro approach. The immunosuppressive effects of PFASs were comparable to those induced by rapamycin, a well-known immunosuppressant. This finding is significant as it indicates that PFASs can have deep effects on immune function, even in some case at low concentrations relevant to human exposure (for adults the median concentration in serum or plasma was 7.7 ng/mL for PFOS, 1.9 ng/mL for PFOA, 0.67 ng/mL for PFHxS and 0.61 ng/mL for PFNA) (EFSA CONTAM PANEL et al. 2020). Our results are also in line with existing epidemiological and animal studies that have shown an inverse relationship between PFASs exposure and antibody levels. For instance, the study by Grandjean et al. (2012) found lower antibody levels in children with higher serum levels of PFASs, which was later confirmed by Kielsen et al. (2016) in a Danish cohort. Additionally, the study by De Guise and Levin (2021) demonstrated a reduction in anti-KLH-specific IgM in mice after PFOA exposure, further supporting the immunotoxic effects of PFASs observed in our study. The reduction of the IgM response to TD is also the hallmark of PFOA immunotoxicity in the study conducted in mice (Taylor et al. 2023). Moreover, a recent in vitro study, conducted on Jurkat T-cells, highlights that PFASs exposure may lead to a decrease in T-cell activation. This reduction could play a role in diminishing the antibody response that relies on T-cell activity (Janssen et al. 2024).

The proposed TI protocol was reproducible and robust, with a consistent and significant increase in the release of IgG and IgM across a large cohort of donors compared to unstimulated controls tested over a 2-year period, and a consistent reduction with rapamycin. Interestingly, the IC_20_ values calculated for the four PFASs on immunoglobulin release revealed higher susceptibility in females, with lower IC_20_ for PFOA, PFNA, and PFHxS compared to males. This finding suggests that NAMs could also capture sex-specific differences. The results obtained with the TI antibody response following PFAS exposure underscore the critical importance of evaluating male and female responses to toxicants independently, particularly when dealing with substances known to act as endocrine modulators, such as PFASs. This distinction is vital because the endocrine system plays a key role in regulating numerous physiological processes, many of which differ significantly between sexes. Failing to account for these differences can lead to incomplete or inaccurate assessments of a toxicant's impact. In the case of PFASs, their ability to interfere with hormonal pathways makes it even more essential to separately examine male and female responses, as the toxicological effects may vary not only in magnitude but also in the underlying mechanisms.

Significant sex-based variations have been documented in the metabolism, excretion, and health effects of PFASs across various species, including humans (Rickard et al. 2023). When examining the effects of PFASs between sexes in both human epidemiological studies and in vivo animal research, a discernible difference in sensitivity between males and females becomes apparent (Fenton et al. 2021). However, with respect to immune system effects, these differences have not been thoroughly quantified in terms of antibody release. Most studies have predominantly focused on factors such as serum concentration, half-life, and hormonal impacts, rather than directly assessing variations in antibody levels between sexes. This research gap underscores the necessity for further investigations to elucidate how these factors may influence the immune response to PFASs in a sex-specific context.

A possible hypothesis to explain the different susceptibility to PFASs between sexes’ exposure could involve hormonal and metabolic differences. Sex hormones, such as estrogens and testosterone, can modulate the immune response (Sciarra et al. 2023). These hormones may also interfere with the kinetics of PFASs, altering their biodistribution, accumulation, and clearance between males and females, differently modulating the immune response. In addition, sex differences may play a significant role in how PFASs bind to specific receptors or activate intracellular signaling pathways. These differences could result from variations in receptor expression, affinity, or downstream signaling mechanisms between males and females. Such disparities may lead to distinct biological effects, with certain toxicants exerting stronger or weaker responses depending on sex. Given the ability of PFASs to modulate endocrine functions, understanding these sex-specific interactions is crucial for accurately assessing their toxicity.

Results highlight the potential of NAMs to provide accurate and relevant data for assessing immunotoxicity. Moreover, the sex-specific effects observed, where female donors appeared to be more affected by some PFASs than males, which may also offer a more nuanced and precise assessment of chemical safety. In this study, some PFASs exhibited non-monotonic concentration responses, which may be attributed to their unique mechanisms of action. These mechanisms could involve varying affinities for cellular targets or receptor-mediated effects that differ across concentrations, leading to maximal inhibition even at the lowest concentration tested. Such patterns have been previously observed in both in vitro and in vivo studies on PFASs compounds. These findings highlight the complexity of PFASs immunotoxicity and suggest that traditional dose–response assumptions may not always apply to this class of chemicals. Understanding these non-linear effects is crucial for refining risk assessment approaches and improving the predictive power of in vitro models.

Overall, our results recapitulate human associations evidence and animal in vivo data, and reinforce the need for stringent regulation of PFASs, given their ability to impair immune function at environmentally relevant concentrations. Moreover, the data supports the validity of the proposed in vitro approach for studying the immunotoxicity of PFASs, offering two relevant models that can bridge the gap between in vitro studies and human health outcomes, that can be applied to other case studies. In developing non-animal methods for immunotoxicity assessment, it is essential to recognize both the advantages and inherent limitations of in vitro models. Despite these promising results, our study has limitations that should be addressed in future research. In particular, the TD antibody production could be further refined by incorporating additional factors known to influence antibody responses, thereby enhancing the relevance and accuracy of the findings. While our approach successfully identified the immunomodulatory potential of PFAS, certain discrepancies with in vivo data, such as the lack of inhibition in the TDAR assay for all compounds, may stem from assay sensitivity and biological complexity. The in vitro TDAR relies on a primary immune response, which may not fully capture subtle differences due to the low frequency of naïve antigen-specific cells. Additionally, in vitro systems lack key physiological components present in vivo, including the endocrine system, microbiome, and immunological organs such as the spleen and lymph nodes, which play a crucial role in antibody production. Although NAMs cannot fully replicate the in vivo environment, refining these models will improve their predictive power. Our study represents an important step in developing an alternative to animal-based TDAR assays, with the goal of increasing sensitivity and specificity for detecting immunotoxic effects. Future efforts will focus on optimizing the in vitro primary antibody response to KLH to better reflect real-world immune responses while maintaining their relevance for regulatory applications.

To conclude, our research demonstrated the feasibility of assessing in vitro both TI and primary TD antibody production, providing a comprehensive understanding of the risks associated with PFASs exposure. The synergistic application of these two methods enables a comprehensive risk assessment by addressing two different aspects of chemical immunotoxicity. This integration not only offers a more accurate and human-relevant evaluation, reducing uncertainties in health risk assessment but also supports more effective regulatory decisions and protective measures for both sexes.

## Supplementary Information

Below is the link to the electronic supplementary material.

Supplementary file1 (DOCX 419 KB)

## Data Availability

Raw data will be provided upon request.
