# Supplementary material for: In vitro approaches to investigate the effect of chemicals on antibody production: the case study of PFASs
Source: Arch Toxicol. 2025 Mar 6;99(5):2075–86. doi: 10.1007/s00204-025-03993-6 (PMC12085377; doi:10.1007/s00204-025-03993-6)
Supplement: Supplementary file 1 — Supplementary file1 (DOCX 419 KB) [file 204_2025_3993_MOESM1_ESM.docx]

***Supplementary Materials***

***In vitro* approaches to investigate the effect of chemicals on antibody production: the case study of PFASs**

**Martina Iulini^1^, Valeria Bettinsoli^1,2^, Ambra Maddalon^1^, Valentina Galbiati^1*^, Aafke W.F. Janssen^3^, Karsten Beekmann^3^, Giulia Russo^4^, Francesco Pappalardo^4^, Stella Fragki^5^, Alicia Paini****^5^, Emanuela Corsini^1^**

^1^Laboratory of Toxicology, Department of Pharmacological and Biomolecular Sciences “Rodolfo Paoletti”, Università degli Studi di Milano, Milan, Italy

^2^Department of Pharmacy, Università degli Studi di Napoli Federico II, Napoli, Italy

^3^Wageningen Food Safety Research (WFSR), Wageningen, The Netherlands

^4^Department of Health and Drug Sciences, Università degli Studi di Catania, Italy

^5^esqLABS GmbH, 26683 Saterland, Germany

* Corresponding Author:

Valentina Galbiati

Laboratory of Toxicology,

Department of Pharmacological and Biomolecular Sciences “Rodolfo Paoletti”

Università degli Studi di Milano

Via Balzaretti 9,

20133 Milan, Italy.

E-mail address: valentina.galbiati@unimi.it


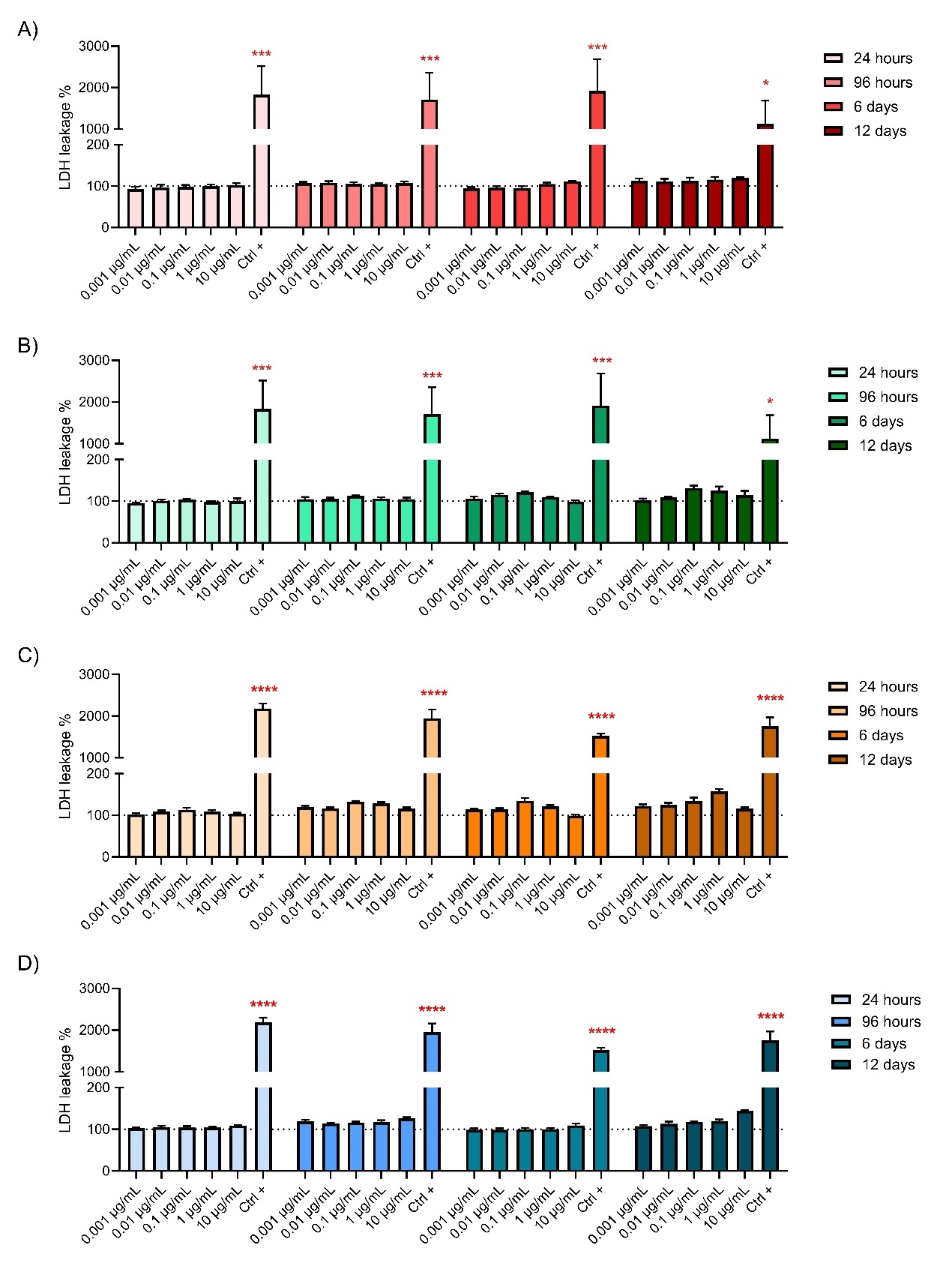


**Figure 1: Effects of PFOA, PFNA, PFHxS and PFOS on cell viability.** PBMCs (1x10^6^ cells/ml) were treated for 24 and 96h, 6 and 12 days with increasing concentrations of PFOA (A), PFNA (B), PFHxS (C) and PFOS (D). The Ctrl + is the LDH positive control present in the CyQUANT™ LDH Cytotoxicity Assay Kit. Results are expressed as percentage (%) of cytotoxicity. Each value represents the mean ± standard error of the mean (SEM), with n = 2 male and 2 female donors pooled together. Statistical analysis was performed using two-way ANOVA, followed by Dunnett’s test. Results were considered significant if p ≤ 0.05, with * p ≤ 0.05 and ** p ≤ 0.001 vs DMSO (represented by the dot line set at 100 %). The same figure is also reported in Corsini et al. (2024).
